# Properties of New Composite Materials Based on Hydroxyapatite Ceramic and Cross-Linked Gelatin for Biomedical Applications

**DOI:** 10.3390/ijms23169083

**Published:** 2022-08-13

**Authors:** Michał Bartmański, Magda Rościszewska, Marcin Wekwejt, Anna Ronowska, Małgorzata Nadolska-Dawidowska, Aleksandra Mielewczyk-Gryń

**Affiliations:** 1 Department of Biomaterials Technology, Faculty of Mechanical Engineering and Ship Technology, Advanced Materials Centre, Gdańsk University of Technology, 80-233 Gdańsk, Poland; 2 Department of Laboratory Medicine, Medical University of Gdańsk, 80-210 Gdańsk, Poland; 3 Department of Solid State Physics, Faculty of Applied Physics and Mathematics, Advanced Materials Centre, Gdańsk University of Technology, 80-233 Gdańsk, Poland

**Keywords:** injectable composite, hydroxyapatite ceramic, gelatin, transglutaminase, biomedical application

## Abstract

The main aim of the research was to develop a new biocompatible and injectable composite with the potential for application as a bone-to-implant bonding material or as a bone substitute. A composite based on hydroxyapatite, gelatin, and two various types of commercially available transglutaminase (TgBDF/TgSNF), as a cross-linking agent, was proposed. To evaluate the impacts of composite content and processing parameters on various properties of the material, the following research was performed: the morphology was examined by SEM microscopy, the chemical structure by FTIR spectroscopy, the degradation behavior was examined in simulated body fluid, the injectability test was performed using an automatic syringe pump, the mechanical properties using a nanoindentation technique, the surface wettability was examined by an optical tensiometer, and the cell viability was assayed by MTT and LDH. In all cases, a composite paste was successfully obtained. Injectability varied between 8 and 15 min. The type of transglutaminase did not significantly affect the surface topography or chemical composition. All samples demonstrated proper nanomechanical properties with Young’s modulus and the hardness close to the values of natural bone. BDF demonstrated better hydrophilic properties and structural stability over 7 days in comparison with SNF. In all cases, the transglutaminase did not lead to cell necrosis, but cellular proliferation was significantly inhibited, especially for the BDF agent.

## 1. Introduction

Long-term implants are fixed in bone, cartilage, or connective tissue in different ways, often determining the primary and long-term stabilization of an implant. Rejection of the implants after surgery is the most fundamental implantation problem. It may be caused by allergy to the implant, inflammation, failure to follow medical recommendations by the patient, or a surgeon’s mistake [1,2]. However, the most common one is a lack of or very weak adhesion between the implant and the bone tissue.

There are many ways to improve bone–implant adhesion; for example, bone cement stabilization is achieved almost exclusively by instantaneously hardening polymers (e.g., PMMA, poly(methyl methacrylate)). The great disadvantage of PMMA is the exothermic hardening reaction, resulting in a rise in temperature and an increased risk of necrosis of adjacent tissue. Therefore, bone-like composite materials used to fix implants have been the objects of research for many years. Hydroxyapatite (HAp) and nanohydroxyapatite (nanoHAp) are the most popular materials or components of material among the apatites. Gelatin, due to its highly biocompatible, unique biological, non-immunogenic, and bioresorbable properties is a frequently applied polymer for biomedical applications. However, its solubility at body temperature (i.e., T = 37 °C) and low biomechanical properties, mean that an additional cross-linking mechanism is required. Therefore, chemical modification and mixing of gelatin with other biomaterials are strategies proposed to improve gelatin’s properties and its stability.

Maji et al. [3] reported a hydroxyapatite–chitosan/gelatin nanocomposite scaffold where glutaraldehyde (GTA) acted as a cross-linking agent for biopolymers. Sharma et al. [4] developed a combination of chitosan, gelatin, alginate, and a nanohydroxyapatite. The obtained composites possessed excellent properties of hydrophilicity and biodegradability. In vitro cell culture studies with seeded osteoblasts on the scaffolds showed good cell viability, proliferation rate, adhesion, and maintenance of the osteoblastic phenotype. Azami et al. [5] designed a nano-structured scaffold using hydroxyapatite and gelatin as its main components. Osteoblast-like cells were seeded on these scaffolds, and their proliferation rate, intracellular alkaline phosphatase (ALP) activity, and ability to form mineralized bone nodules were comparable with those of osteoblasts grown in cell culture flasks. Tavakol et al. [6] developed a nanohydroxyapatite/gelatin composite with higher osteoblast attachment and proliferation than micro-sized hydroxyapatite, and a shorter culturing period and lower cell seeding density compared with pure gelatin. Its biocompatibility was demonstrated with the alizarin red test on Days 14 and 21. The calcium deposits were significantly elevated, and there was an increase in the markers expressed in osteoblast differentiation on the 21st day of osteoblast culture directly on the composite in vitro. Lee et al. [7] proposed a synthetic bone scaffold based on hydroxyapatite–gelatin–calcium silicate. Chen et al. [8] investigated the gelatin methacrylate (GelMA), a photo cross-linkable and biocompatible hydrogel, together with nanohydroxyapatite. Nouri-Felekori et al. [9] developed a composite scaffold of CaP–gelatin. Mixed forms of CaP particles, in the shape of whiskers or spherulites, were incorporated into the gelatin, influencing the improvement in the mechanical properties of the implant. Ferreira et al. [10] developed novel cell aggregate-loaded macroporous scaffolds combining the osteoinductive properties of titanium dioxide (TiO_2_) with hydroxyapatite–gelatin nanocomposites for regeneration of craniofacial defects. Hamlekhan et al. [11] obtained nanocomposite scaffolds by using poly(ε-caprolactone), cross-linked gelatin, and nanoparticles of hydroxyapatite, where glutardaldehyde was used as a cross-linking agent.

Composites consisting of hydroxyapatite, gelatin, and additives can be also used as an injectable biomaterial graft, which serves as a filler. For example, Uskokovic et al. [12] investigated antimicrobial hydroxyapatite–gelatin–silica composite pastes with tunable setting properties. Sari et al. [13] used a hydroxyapatite–gelatin–hydroxypropyl methylcellulose composite as an injectable bone substitute with alendronate variation for osteoporotic bone. Moreover, Maulida et al. [14] synthesized and characterized an injectable bone substitute paste based on hydroxyapatite, gelatin, and streptomycin. In order to obtain an injectable composite, it is necessary to use a gel as the matrix material with a thickening ability caused by high viscosity, leading to in situ polymerization after being injected. For example, in [14], hydroxypropyl methylcellulose (HPMC), which is widely used in the food industry, was used as a suspending agent. A stable form of calcium phosphate, hydroxyapatite, embedded in a polymeric gelatin matrix was proposed to provide adequate mechanical properties and biocompatibility, and to ensure proper osteointegration. 

The main problem with composites based on biopolymers is their biostability, which is significantly influenced by the cross-linking process. Ongoing research is focused on developing a novel and effective method of cross-linking the polymer matrix. Chemical cross-linking is a relatively popular and available method; however, most of the cross-linking agents (such as glutaraldehyde, formaldehyde, tetramethylethylenediamine, polyethylene glycol, and epoxy compounds) have a toxic effect on human cells and are not environmentally friendly [15], which is a significant disadvantage of such solutions in medical applications. Moreover, they are usually relatively expensive, which increases the costs of their future use as biomaterials. Hence, in this study, transglutaminase, an enzyme mainly used in the food industry [16], was proposed as alternative cross-linking agent. This enzyme has been used successfully for gelatin and the results have been promising. [17]. The combination of hydroxyapatite, gelatin, and transglutaminase for implant applications has been never reported before. We assumed that the use of enzymatic cross-linking of gelatin would improve the biostability of the proposed hydroxyapatite composite as well as its overall cellular response. In addition, injectability tests for this type of material are performed rarely or not at all [3,12]. Due to the nature of the material’s application proposed in the present study, performing injectability tests was necessary.

The aim of this research was to determine the effect of various manufacturing parameters of a new composite material based on hydroxyapatite ceramics, a natural polymer (gelatin), and different cross-linking agents on the microstructure, nanomechanical properties, degradation, application properties (injectability), wettability, and in vitro biological response to osteoblast cells.

## 2. Results and Discussion

**Figure 1:**
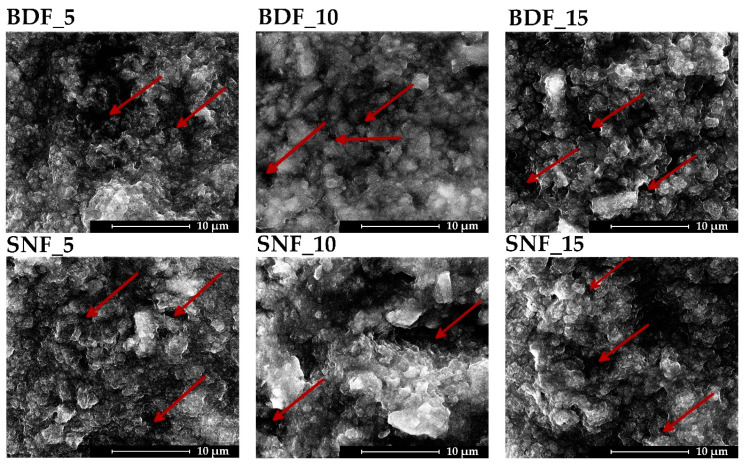
SEM images of the surface of the composite materials (BDF and SNF indicate two types of transglutaminases; 5, 10, and 15 indicate the wt% content of transglutaminases (per gelatin mass)); arrows indicate the pores.

Figure 1 presents the microstructure of the obtained composite materials. No significant effect of the content and type of transglutaminase used on the surface topography was observed. The microstructure consisted of hydroxyapatite particles suspended in cross-linked gelatin. The obtained specimens were characterized mainly by their macroporosity (pore size below 10 μm) and extensive surface topography, which should ensure adequate osteoconductive properties [17]. A similar cluster-like morphology with many fine particles was obtained by Sharma et al. [4], where the high porosity was due to the bonding between the gelatin molecules and the HAp nanopowder.

**Figure 2:**
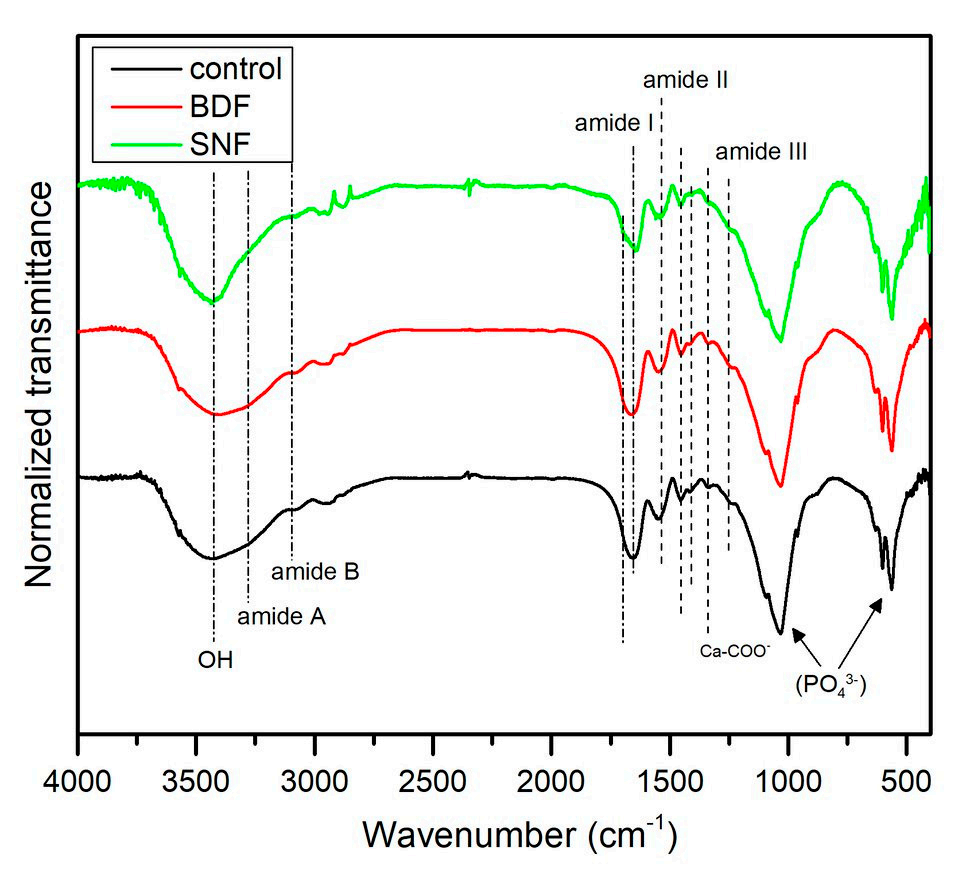
FTIR spectra of HAp composites prepared with BDF- and SNF-cross-linked gelatin and without gelatin cross-linking (control).

Figure 2 presents the representative spectra of HAp composites prepared with BDF- and SNF-cross-linked gelatin and without gelatin cross-linking. The pronounced bands observed in the low wavenumber region, from 450 cm^−1^ to 1020 cm^−1^, are characteristic of HAp and correspond to the vibrations of PO_4_^3−^ [18].

Gelatin showed typical bands at 1635, 1535, and 1245 cm^−1^, which indicate amide I (C=O stretching vibration, hydrogen bonding coupled with COO^–^), amide II (bending vibration of N-H groups and stretching vibration of the C–N group), and amide III bands (in-plane vibration of C–N and N–H groups of bound amide or vibrations of the CH_2_ groups of glycine) [18,19]. Moreover, in all spectra, a band appeared at ~1345 cm^−1^, which indicated the formation of a chemical bond between the carboxyl group from gelatin and a Ca^2+^ ion from HAp [20]. Generally, all spectra looked quite similar, which indicates that the chemical composition of gelatin was not remarkably changed after cross-linking with various agents [21].

**Figure 3:**
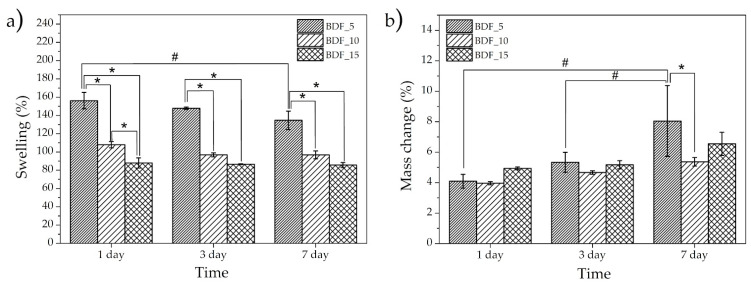
Degradation behavior: swelling (**a**) and mass change (**b**) of composite materials cross-linked with BDF after the immersion in PBS and being stored at 37 °C (n = 3; * significantly different by transglutaminase concentration, ^#^ significantly different by degradation time according to one-way ANOVA followed by Bonferroni’s multiple comparison test, *p* < 0.05).

The swelling ratio at different times was calculated to determine the water absorption capacity, while a weight loss experiment allowed us to investigate the structural stability of the composite (Figure 3). Further, the composite cross-linked using SNFs disintegrated itself after 24 h; thus, the use of this cross-linking agent was not effective. The specimens with the lowest content of BDF transglutaminase (BDF_5) showed the highest swelling tendency (~140–150%) and an increase in BDF content resulted in a decrease in water absorption (~100–110% and ~85–88%, respectively, for BDF_10 and BDF_15). This might be related to the lower binding tightness of gelatin caused by the lower content of the cross-linking agent, leading to a higher swelling ratio. This capacity indicated that the composite could fill bone defect areas in the presence of body fluids [14]. Similarly, in [16], the water adsorption of hydroxyapatite/gelatin composites reached 154–203% after 24 h of incubation. A slight downward trend across the incubation time could also be noticed, which was more visible for a lower transglutaminase content, which may have been due to weight loss. Furthermore, an increase in the cross-linking agent concentration resulted in reduced swelling of the sample.

It was observed that the samples degraded with incubation time. The greatest mass loss (~8% after 7 days) was found for the lowest concentration of BDF (Figure 3b). However, no statistically significant changes in the medium and high concentrations of BDF content were observed (Figure 3b). These observations are comparable with those of [14], where the authors also reported the stability of an injectable bone substitute paste based on hydroxyapatite, gelatin, and streptomycin after 7 days of an acid test.

Biomaterials’ degradation is very important for their clinical application because it allows us to avoid future reoperation and enables cyclic osteoremodeling of bone tissue [22]. Both components of the obtained composite used here are biodegradable in the human body. Hydroxyapatite is one of the most stable calcium phosphate-based materials, as it is only slightly soluble in water solutions and, under physiological body conditions, has a relatively low degradation ability due to numerous dissolution and precipitation processes [23]. The mechanism of its biodegradation behavior, despite numerous studies, is not fully known, but is clear that apart from the abovementioned processes, it is also mediated by osteoclast cells and macrophages [24]. It has been shown that hydroxyapatite degradation depends on various factors, including the Ca/P ratio, purity, microstructure, place of implantation, and body condition [25]. Gelatin, as a natural protein derivative obtained by collagen hydrolysis and as un-cross-linked material, dissolves quickly in a water solution. However, after the cross-linking process, gelatin material exhibits better hydrolytic resistance [26]. Significant differences in swelling and mass change relative to degradation time for samples of the same group were observed only for BDF_5: between 1 and 7 days for swelling, and between 1 and 3 and 1 and 7 days for mass change.

**Figure 4:**
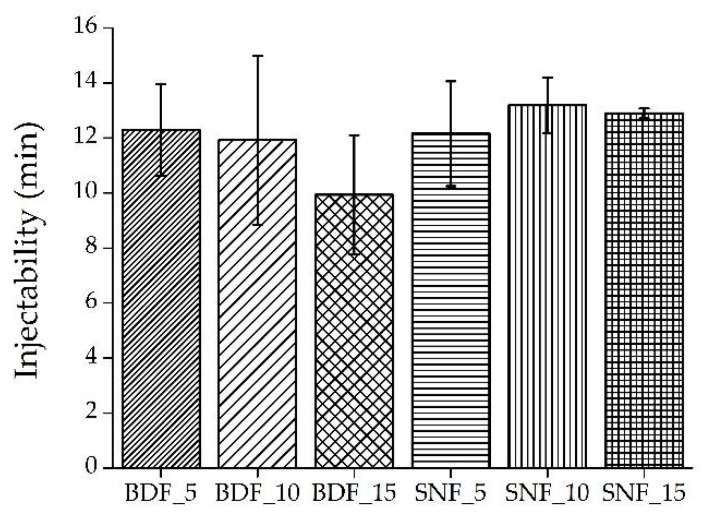
Injectability of a paste of the tested composite (n = 3).

The injectability results are presented in Figure 4. Injectability is affected by the solvent and solute of the composite, the viscosity of the composite, and the syringe diameter. The addition of fillers leads to a decrease in the injectable time and, in a clinical setting, resulted in a shortened operation time [27]. 

As presented in Figure 4, in all cases the injectability varied between 8 and 15 min. In comparison, Maulinda et al. [12] also measured the injectability of a bone substitute paste based on hydroxyapatite, gelatin, and streptomycin. The results showed that during 2 min, about 97% of the paste was injected. 

**Figure 5:**
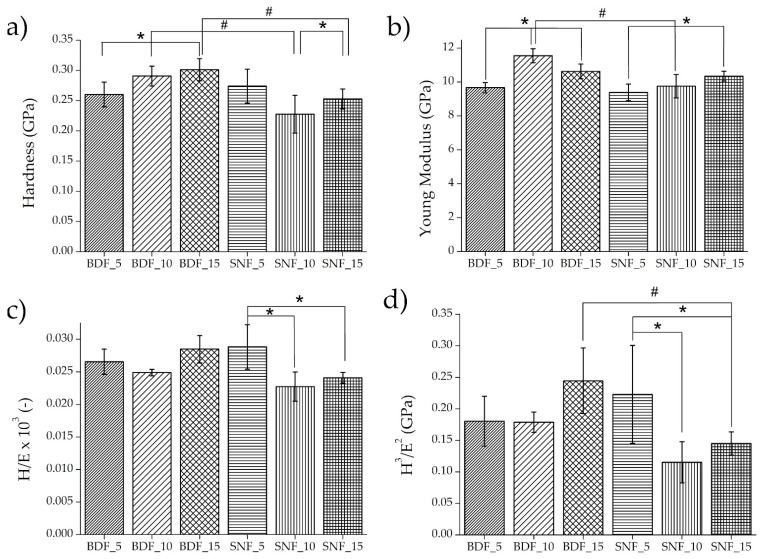
Nanomechanical properties: (**a**) hardness; (**b**) Young’s modulus; (**c**) H/E ratio, and (**d**) H^3^/E^2^ ratio for composite materials (n = 10; * significantly different by transglutaminase concentration, ^#^ significantly different by transglutaminase’ type according to one-way ANOVA followed by Bonferroni’s multiple comparison test, *p* < 0.05).

For implant materials, the mechanical properties of biomaterials should approximate the mechanical properties of the tissues surrounding the implant. In the case of the tested materials, the mechanical behavior of bone tissue should be the reference. Nanoindentation results of the tested specimens are presented in Figure 5. In the case of specimens BDF_10–15, Young’s modulus was 10.6–11.6 GPa, respectively, and these values were equal to those of cortical and trabecular bone lamellae measured by nanoindentation in the human femur (Figure 5b) [28]. However, for all tested composites, the hardness (Figure 5a) was close to the values measured by the nanoindentation technique in the human femur and varied between 0.23 and 0.76 GPa [28]. On the other hand, there are studies where much lower values of Young’s modulus (3.5–4.7 GPa) have been obtained, which could be related to a different hydroxyapatite/gelatin ratio, or the lack of an additional cross-linking agent [29].

The mechanical properties such as the elastic strain to failure of the materials (H/E) and the resistance of a material to plastic deformation (H^3^/E^2^) were also tested using the nanoindentation technique [30,31]. The H/E ratio characterizes the resistance of the material to elastic deformation, and the H^3^/E^2^ ratio allows us to estimate the material’s ability to dissipate the energy of plastic deformation during loading [32]. Both ratios can approximately characterize the wear resistance of a material. An improvement in both coefficients was obtained for BDF_15 and SNF_5 (Figure 5c,d). Because the implant materials are subject to continuous stresses, the increase in the determined coefficients indicates the better applicability of these two materials.

**Figure 6:**
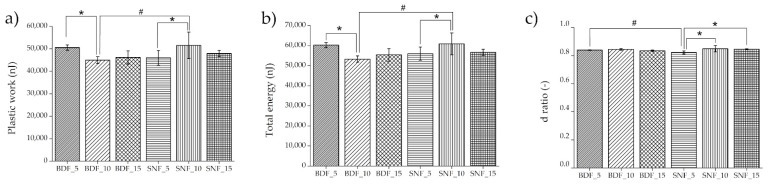
Nanoindentation energy properties: (**a**) plastic energy, (**b**) total energy, and (**c**) d ratio (plastic energy/total energy) for composite materials (n = 10, * significantly different by transglutaminase concentration, ^#^ significantly different by transglutaminase type according to one-way ANOVA followed by Bonferroni’s multiple comparison test, *p* < 0.05).

During the nanoindentation measurements, three types of energy were registered: the total energy of nanoindentation was found under the loading curve, the elastic energy was located under the unloading curve, and the difference between the total and elastic energy was the plastic energy. The energy values obtained by nanoindentation tests can be used to analyze the mechanical behavior of the tested material. It has been shown that an increase in elastic energy can mean better resistance to cracking. The value of the ductility index can predict the material volume/bulk fracture behavior of the tested materials compared with similar fractures generated by nanoindentation stresses [33]. The nanoindentation energy properties are presented in Figure 6.

**Figure 7:**
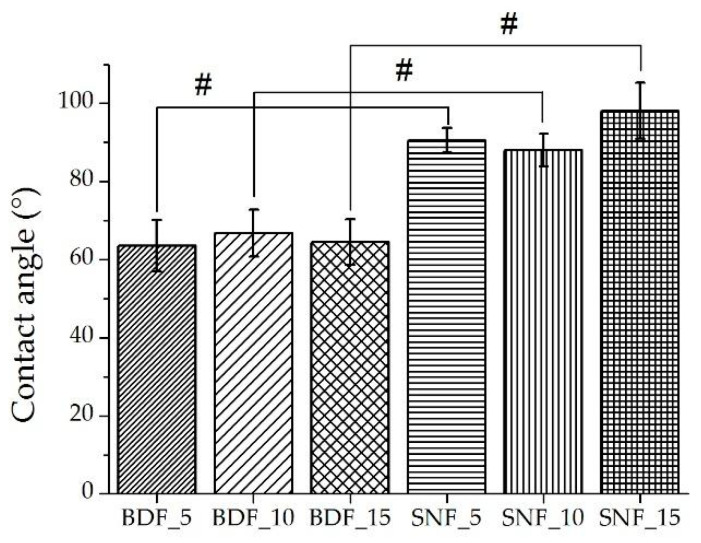
Water contact angle at room temperature for composite materials (n = 3, ^#^ significantly different by transglutaminase’ type according to one-way ANOVA followed by Bonferroni’s multiple comparison test, *p* < 0.05).

The contact angles are depicted in Figure 7. The results for BDF_5–15 and SNF_5–15 indicate that there was no effect of cross-linking agent content on the contact angle values for the same group of materials (Figure 7). A significant increase in the contact angle value was observed when changing the type of cross-linking agent from BDF to SNF, and it was about 40% higher for SNF compared with BDF. For specimens cross-linked with SNF transglutaminase, hydrophilicity/hydrophobicity limit values were obtained. The effect of contact angle on in vitro biological responses has been demonstrated in numerous studies [34,35,36]. It has been shown that the optimum wettability angle for osteoblast proliferation is in the range of 35–85°; more precisely, it is 55° [37]. The results showed that the contact angle obtained for BDF samples is ~65° (Figure 7). Therefore, the use of BDF modification makes the material more biocompatible. 

**Figure 8:**
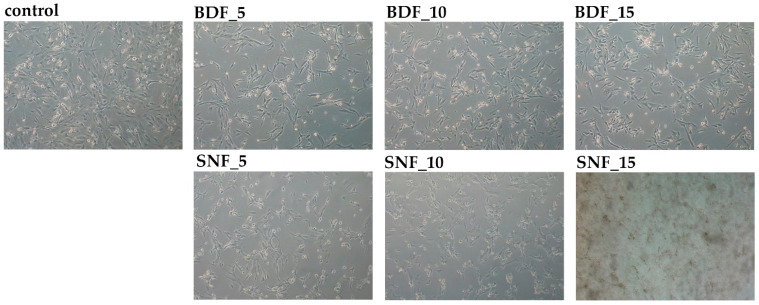
Effects of BDF and SNF on the morphology of hFOB 1.19 cells (magnification 100×). Data are representative of 4 experiments.

Finally, the biocompatibility of the proposed materials was determined. The hFOB 1.19 cell culture model was used for that purpose. The morphology of the cells was changed in response to BDF/SNF exposure. Both BDF and SNF at a 5% concentration brought about a loss of cellular extensions and intercellular connections. Notwithstanding, the cells treated with SNF did not create blebs, and less malformation of the cell membranes was noticed compared with BDF (Figure 8). These alterations were not significantly changed by increasing the concentration of the tested transglutaminases (Figure 8). It was shown that SNF disintegrates after 24 h of incubation in a PBS buffer.

**Figure 9:**
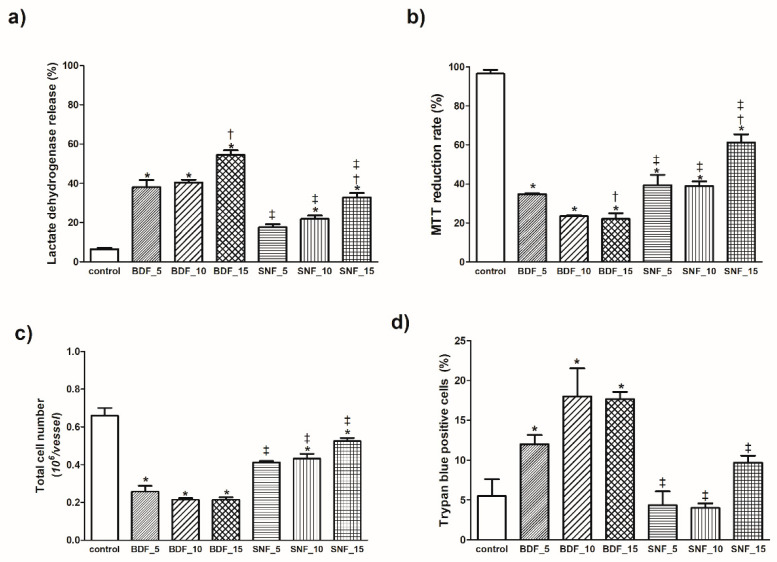
The effects of BDF and SNF on (**a**) lactate dehydrogenase release, (**b**) MTT reduction rate, (**c**) total cell number, (**d**) trypan blue-positive hFOB 1.19 cells. Data are the means ± SEM from n = 4–6 independent cell culture preparations. Significantly different from the respective controls: * *p* < 0.05, BDF_5; ^†^ *p* < 0.05, BDF; ^‡^ *p* < 0.05.

**Figure 10:**
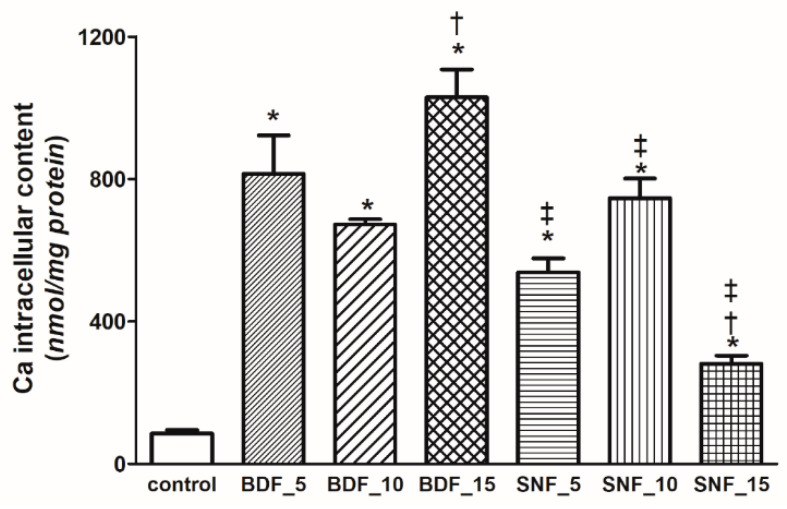
The effects of BDF and SNF on intracellular Ca^2+^ levels in hFOB 1.19 cells. Data are the means ± SEM from n = 4–6 independent cell culture preparations. Significantly different from respective controls: * *p* < 0.05, BDF_5; ^†^ *p* < 0.05, BDF; ^‡^ *p* < 0.05.

**Figure 11:**
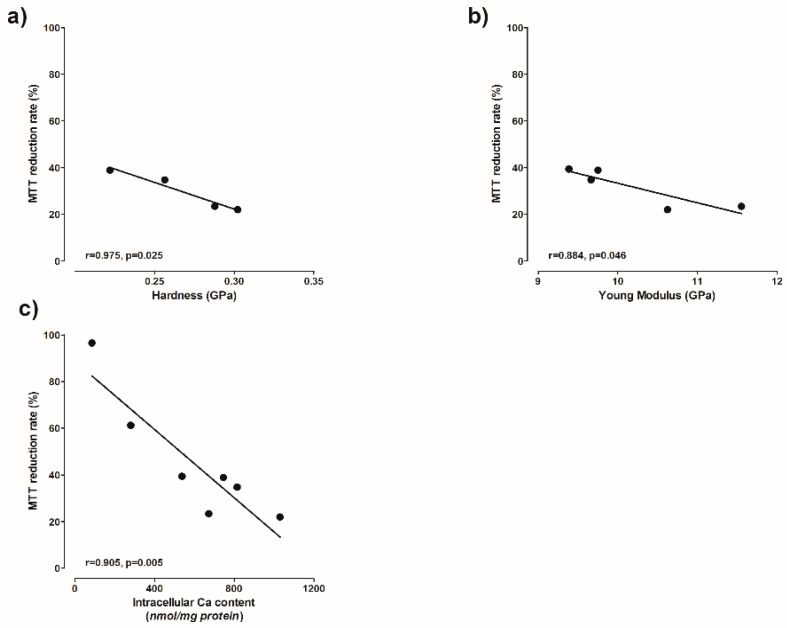
The correlations between: (**a**) reduction in MTT and hardness, (**b**) reduction in MTT and Young’s modulus, and (**c**) reduction in MTT and intracellular Ca content in hFOB 1.19 cells. Data were taken from Figure 5, Figure 9, and Figure 10.

Culture of the cells with BDF/SNF for 24 h did not cause cell death. The SNF at concentrations of 5% and 10% did not lead to cell necrosis because the LDH release rate was not meaningly enhanced in the tested cells (Figure 9a). Moreover, the cell morphology was not characterized by the necrotic phenotype; for example, cytoplasmic blebs were not noticeable. On the other hand, cellular proliferation was significantly inhibited, because the MTT reduction rate was about 40% in these conditions (Figure 9b). Moreover, the total cell number decreased by about 36%, and fraction of damaged cells was not changed compared with the control, which agrees with the MTT and LDH results (Figure 9c,d). An increase in this transglutaminase in the composite to 15% increased LDH release by 33% but the cellular proliferation was about 62% under these conditions (Figure 9). The cell number decreased by only 20% with 15% SNF (Figure 9c). Transglutaminase used in BDF modification has proven to increase Ca deposition under appropriate cell culture conditions. However, this concentration leads to excessive intracellular Ca^2+^ content, which promotes cell death. Thus, the concentration of transglutaminase used is too high despite its mechanical advantages. This enzyme catalyzes the transamidation reaction between the amino group of lysine residues and the carboxamide group of glutamine residues, thus forming gelatin hydrogels under safe conditions. This indicates that cross-linking of this transglutaminase decreased the Ca^2+^ release from hydroxyapatite. The intracellular level of this cation was lowest in the cells treated with 15% SNF (Figure 10). The viability of the cells depends the intracellular Ca level (Figure 11c). The higher the calcium level, the lower the viability (Figure 11c). A high concentration of intracellular Ca^2+^ promotes cell death by the apoptotic pathway. BDF at concentrations of 5 and 10% led to about 65 and 76% cellular growth inhibition, respectively (Figure 9b,c). The LDH release under these conditions was about 35% (Figure 9a). Moreover, the fraction of damaged cells was significantly increased by the presence of 5 and 10% BDF (Figure 9d). An increase in the transglutaminase content to 15% caused 50% cell death, as measured by the LDH release, and almost 80% inhibition of cellular proliferation (Figure 9a–c). Moreover, the cellular mitochondrial activity also indirectly depends on the hardness and Young’s modulus (Figure 11a,b). 

Despite the promising results of the novel biocomposite formulation, this study is not without limitations. First, all the experiments were conducted using one type of hydroxyapatite powder with a certain particle size and shape. It is likely that changing these properties of the powder will affect both the physico-mechanical and biological results. Second, the screening cytotoxicity tests and degradation behavior experiments do not fully reflect the complex processes of in vivo conditions. In the case of degradation, the effect of active bioresorption caused by living cells was missing, while in terms of cytocompatibility, the main problem was the lack of circulation of body fluids, which undoubtedly affects ion exchange and has an influence on the cellular response. Thirdly, the mechanical properties of the composite were evaluated by the microindentation method; however, this method does not fully represent the behavior of the biomaterial under conditions of the variable and cyclic forces occurring in the human body. Considering the points above, future studies will be needed in order to accurately characterize the novel composite in terms of the following properties, among others: adhesive abilities, full degradation time under in vivo conditions, compressive and flexural strength under conditions similar to the living organism, and the complete cellular response in in vivo studies, especially in terms of stimulating osteogenic differentiation. Taken together, the results obtained here, despite the limitations listed above, allow us to select the initial and optimal composition of a novel promising composite for biomedical applications, which can now be investigated further.

## 3. Materials and Methods

### 3.1. Preparation of the Specimens

To make the composite, 4 g of gelatin was dissolved in 9 mL of distilled water at 50 °C and stirred by a magnetic stirrer at 200 rpm for 5 min. Next, 8 g of hydroxyapatite powder with an average grain size of <200 µm (Sigma Aldrich, St. Louis, MO, USA) was added while continuously mixing at 50 °C. Two types of transglutaminase, namely BDF (Probind TXo, BDF Natural Ingredients S.L) and SNF (TG-S-NF, Ajinomoto Co., Tokio, Japan), which had previously been dissolved in 1 mL of distilled water, were added to obtain a composite paste at 5, 10 or 15 wt% (per gelatin mass). After 10 s of intense mixing, the composite was placed in appropriately prepared polymer molds 20 mm in diameter and 4 mm in height or, for the injectability studies, in syringes 10 mm in diameter and 20 mL in volume. The samples, after curing, were removed from the molds and left for 5 days at room temperature to evaporate water. 

### 3.2. Microstructural and Chemical Composition Examinations

The microstructure of the composite materials was evaluated using scanning electron microscopy (SEM, FEI Company, Hillsboro, OR, USA). The observations were carried out on specimens sputter-coated with gold. The Fourier-transform infrared (FTIR) spectra were registered by a spectrophotometer (Perkin Elmer Frontier, Poznań, Poland) at a resolution of 2 cm^−1^ within the spectral range from 400 to 4000 cm^−1^. Pressed and mixed samples with KBr allowed for measurement in transmittance mode.

### 3.3. Degradation Behavior

The composite materials were placed in 10 mL of a PBS solution (Merck, Darmstadt, Germany) and incubated at 37 °C for 1, 3, and 7 days (n = 3). Next, the mass changes were weighed and relative changes in the weight were calculated. In addition, after immersion, the samples were dried for 7 days at room temperature to evaporate water.

### 3.4. Injectability Tests

Injectability tests were performed using an automatic syringe pump (World Precision Instruments, Sarasota, FL, USA). For this, 10 g of the composite paste was added to identical syringes 20 mL in volume and 10 mm in diameter. Injection was tested at a constant linear speed of 100 µm/min. The injection time was measured until the paste was not injectable (n = 3). 

### 3.5. Mechanical Properties

To determine the mechanical properties, a nanoindenter (NanoTest Vantage, MicroMaterials, Wrexham, UK) equipped with a standard Berkovich tip was used. Ten independent measurements were performed on each sample. The maximum load of 5 N was reached after 15 s of loading time, followed by 5 s of holding before unloading. The distance between each indents was 100 µm. After each indentation test, temperature drift was allowed for 10 s. The hardness, plastic work, elastic work, and reduced Young’s modulus, were determined using the Oliver–Pharr method [38]. In order to calculate the Young’s modulus values, Poisson’s ratio for the tested composite materials was assumed to be equal to 0.28.

### 3.6. Wettability Tests

The wettability studies were evaluated via an optical tensiometer (Attention Theta Life, Biolin Scientific, Espoo, Finland) using a falling water drop method at room temperature with the volume of the drop being equal to 250 μL (n = 3).

### 3.7. In Vitro Cytotoxicity Experiments

The screening experiments on the cytotoxicity of BDF and SNF were conducted on the human hFOB 1.19 cell line (RRID: CVCL 3708), obtained from ATCC (American Type Culture Collection). The cell line is used as a model of human osteoblasts in in vitro studies. Cells were cultured in a 1:1 mixture of Ham’s F12 Medium and Dulbecco’s Modified Eagle’s Medium (without phenol red), supplemented with 0.3 mg/mL G418 and 10% FBS. The culture was grown at 34 °C in a humidified atmosphere of 5% CO_2_ and 95% air. The number of passages before each experiment was 2. 

### 3.8. Cell Viability Assays

**Scheme 1:**
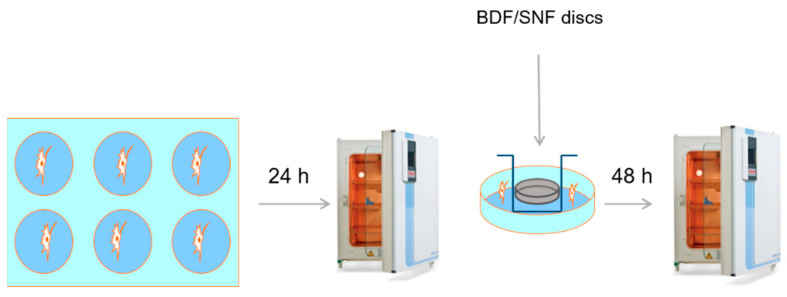
Samples of the materials (20 mm in diameter and 4 mm in height) were placed on inserts 3 cm in diameter with a semipermeable bottom and a 0.4 µM pore size. Each insert was placed into the wells in the 6-well plate with the hFOB 1.19 cells. The culture was continued for the next 48 h.

The cytotoxicity of BDF and SNF was measured by the MTT cytotoxicity test and the LDH release assay. Moreover, the total cell number was estimated with trypan blue dye. The hFOB 1.19 cells were seeded at a density of 3 × 10^5^ cells per well of a 6-well plate. The cells were between Passages 5 and 8. The cells were preconditioned for 24 h. Samples of the materials (20 mm in diameter and 4 mm in height) were placed on inserts 3 cm in diameter with a semipermeable bottom and a 0.4 µM pore size. Each insert was placed into the wells in the 6-well plate with the hFOB 1.19 cells. The culture was continued for the next 48 h (Scheme 1). Following that, the cytotoxicity tests were conducted.

*LDH release assay*. The medium of the cells was used for the LDH ((S)-lactate:NAD^+^oxidireductase (lactate dehydrogenase, LDH, EC 1.1.1.27)) release assay. This enzyme is located in the cytoplasm; therefore, its release to the culture medium is elevated during necrotic cell death. For this enzyme assay, the activity was determined by the direct measurement of NADH oxidation at 340 mm. LDH data were expressed as a percentage of the total LDH released from non-treated cells.

*MTT assay*. The MTT assay measured the activity of mitochondrial dehydrogenases. This involves the conversion of 3-(4,5-dimethylthiazol-2-yl)-2,5-diphenyltetrazolium bromide, which is yellow, to a blue formazan product. This reaction takes place only in living cells. 

After 48 h of BDF and SNF treatment, fresh medium with 0.60 mmol/L MTT was added to the cells. The culture was continued for the next 4 h. The production of formazan was quantified by spectrophotometric measurement at 570 nm [39]. The viability was presented as relative values against the non-treated controls.

*Trypan blue assay*. This test aimed to estimate the total cell number and the number of damaged cells. A solution of trypan blue dye was added to 0.02 mL of the cell suspension at a 0.4% (w/v) concentration. The normal, damaged, and non-viable cells accumulated in the dye were counted under a light microscope after 2 min of incubation using a Fuchs-Rosenthal hemocytometer. 

### 3.9. Determination of the Intracellular Calcium Level 

For the calcium assessment, the cells were grown on plates 3 cm in diameter with and without BDF and SNF. After 48 h, the cells that remained attached to the plates were gently washed with ice-cold PBS. Following that, the cells were deproteinized with ice-cold 4% HCLO_4_ acid. Ca was measured in the supernatant using Asrenaso III dye. Absorbance was measured at 650 nm. The Ca concentrations were quantified on the basis of a calibration curve with CaCl_2_ as the standard. 

### 3.10. Statistical Analysis

An appropriate number of repetitions for each group of samples was used. The results are presented as means ± standard deviations (SD) and the obtained relationships were analyzed using one-way ANOVA using the Bonferroni *t*-test, with the statistical significance set at *p* < 0.05. The Shapiro–Wilk test was used to assess the normal distribution of the data. For biological assessment of the human osteoblasts, statistical analyses were carried out using GraphPad Prism 5 (GraphPad software, La Jolla, CA, USA).

For the viability assays, statistical analyses were carried out using GraphPad Prism 5 (GraphPad software, La Jolla, CA, USA). Data are the means ± SD from 3–5 independent cell culture preparations. The assessment of normality of the data was carried out by the Shapiro–Wilk normality test. The two groups were compared by the non-parametric Mann–Whitney test, with *p* < 0.05 being considered to be statistically significant. 

## 4. Conclusions

In this study, a new formulation and fabrication method for a biocompatible and injectable composite with potential for application as a bone-to-implant bonding material or as a bone substitute were evaluated. A composite based on hydroxyapatite, gelatin, and two types of transglutaminase (BDF/SNF) as a cross-linking agent was proposed. In all cases, a composite paste was successfully obtained. 

The type of transglutaminase did not significantly affect the surface topography, chemical composition, or application properties such as injectability. Regardless of the cross-linking agent, all samples demonstrated very good nanomechanical properties, with Young’s modulus and hardness close to the values of natural bone. 

The cytotoxicity studies proved the influence of the type and rate of transglutaminase on the cellular response. In all cases, the transglutaminase did not lead to cell necrosis, but cellular proliferation was significantly inhibited, especially by BDF. However, the poor stability of composites containing SNF proved that this cross-linking agent is not effective. All BDF samples were characterized by their water absorption capacity. Moreover, the wettability measurements revealed that the addition of BDF resulted in better hydrophilic properties compared with specimens cross-linked with SNF transglutaminase, where hydrophilicity/hydrophobicity limit values were obtained.

The present study proved that the new formulation of a biocompatible and injectable composite can be characterized from the perspective of application and its mechanical properties. In order to improve the effects on the cellular response, further studies are needed to carefully evaluate an adequate cross-linking range.

## 5. Patents 

The results presented in this study are part of the patent application “Method of preparation of a ceramic–polymer composite”, number P.439494.

## Data Availability

The data presented in this study are available on request from the corresponding author.

## References

[1] Neoh K.G., Hu X., Zheng D., Kang E.T. (2012). Biomaterials Balancing osteoblast functions and bacterial adhesion on functionalized titanium surfaces q. Biomaterials.

[2] Chaturvedi T. (2013). Allergy related to dental implant and its clinical significance. Clin. Cosmet. Investig. Dent..

[3] Maji K., Dasgupta S., Kundu B., Bissoyi A. (2015). Development of gelatin-chitosan-hydroxyapatite based bioactive bone scaffold with controlled pore size and mechanical strength. J. Biomater. Sci. Polym. Ed..

[4] Sharma C., Dinda A.K., Potdar P.D., Chou C.F., Mishra N.C. (2016). Fabrication and characterization of novel nano-biocomposite scaffold of chitosan-gelatin-alginate-hydroxyapatite for bone tissue engineering. Mater. Sci. Eng. C.

[5] Azami M., Tavakol S., Samadikuchaksaraei A., Hashjin M.S., Baheiraei N., Kamali M., Nourani M.R. (2012). A porous hydroxyapatite/gelatin nanocomposite scaffold for bone tissue repair: In vitro and in vivo evaluation. J. Biomater. Sci. Polym. Ed..

[6] Tavakol S., Azami M., Khoshzaban A., Kashani I.R., Tavakol B., Hoveizi E., Sorkhabadi S.M.R. (2013). Effect of laminated hydroxyapatite/gelatin nanocomposite scaffold structure on osteogenesis using unrestricted somatic stem cells in rat. Cell Biol. Int..

[7] Lee D.J., Padilla R., Zhang H., Hu W.S., Ko C.C. (2014). Biological assessment of a calcium silicate incorporated hydroxyapatite-gelatin nanocomposite: A comparison to decellularized bone matrix. BioMed Res. Int..

[8] Chen X., Bai S., Li B., Liu H., Wu G., Liu S., Zhao Y. (2016). Fabrication of gelatin methacrylate/nanohydroxyapatite microgel arrays for periodontal tissue regeneration. Int. J. Nanomed..

[9] Nouri-Felekori M., Mesgar A.S.M., Mohammadi Z. (2015). Development of composite scaffolds in the system of gelatin-calcium phosphate whiskers/fibrous spherulites for bone tissue engineering. Ceram. Int..

[10] Ferreira J.R., Padilla R., Urkasemsin G., Yoon K., Goeckner K., Hu W.S., Ko C.C. (2013). Titanium-enriched hydroxyapatite-gelatin scaffolds with osteogenically differentiated progenitor cell aggregates for calvaria bone regeneration. Tissue Eng. -Part A.

[11] Hamlekhan A., Moztarzadeh F., Mozafari M., Azami M., Nezafati N. (2011). Preparation of laminated poly(ε-caprolactone)-gelatin-hydroxyapatite nanocomposite scaffold bioengineered via compound techniques for bone substitution. Biomatter.

[12] Uskoković V., Ghosh S., Wu V.M. (2017). Antimicrobial hydroxyapatite-gelatin-silica composite pastes with tunable setting properties. J. Mater. Chem. B.

[13] Sari N., Putra A.P., Siswanto, Pradipta M.F.F., Hikmawati D. (2020). Hydroxyapatite-gelatin-HPMC composite as injectable bone substitute with alendronate variation for osteoporotic bone. AIP Conf. Proc..

[14] Nur Maulida H., Hikmawati D., Budiatin A.S. (2015). Injectable Bone Substitute Paste Based on Hydroxyapatite, Gelatin and Streptomycin for Spinal Tuberculosis. J. Spine.

[15] Maitra J., Kumar Shukla V. (2014). Cross-linking in Hydrogels—A Review. Am. J. Polym. Sci..

[16] Ribeiro W.O., Ozaki M.M., Santos M., Rodríguez A.P., Pflanzer B., Aparecida M., Pollonio R. (2021). Interaction between papain and transglutaminase enzymes on the textural softening of burgers. Meat Sci..

[17] Sabaghi M., Joly C., Adt I., Ozturk K., Cottaz A., Degraeve P. (2022). Food Bioscience Effect of crosslinking by microbial transglutaminase of gelatin films on lysozyme kinetics of release in food simulants. Food Biosci..

[18] Zhang Z., Li K., Zhou W., Gu J., Liu Y., Han C.C. (2020). Factors Influencing the Interactions in Gelatin/Hydroxyapatite Hybrid Materials. Front. Chem..

[19] Golubevas R., Stankeviciute Z., Zarkov A., Golubevas R. (2020). Acrylate–gelatin–carbonated hydroxyapatite (cHAP) composites for dental bone-tissue applications. Mater. Adv..

[20] Azami M., Rabiee M., Moztarzadeh F. (2010). Glutaraldehyde Crosslinked Gelatin / hydroxyapatite Nanocomposite Scaffold, Engineered via Compound Techniques. Plym. Compos..

[21] Dae H.L., Yoshinori A., Nobuhiko Y., Asato T., Tae W.K., Atsushi T. (2019). Cellular Orientation on Repeatedly Stretching Gelatin Hydrogels with Supramolecular Cross-Linkers. Polymers.

[22] Narkar A.R., Tong Z., Soman P., Henderson J.H. (2022). Smart biomaterial platforms: Controlling and being controlled by cells. Biomaterials.

[23] Sathiyavimal S., Vasantharaj S., LewisOscar F., Selvaraj R., Brindhadevi K., Pugazhendhi A. (2020). Natural organic and inorganic–hydroxyapatite biopolymer composite for biomedical applications. Prog. Org. Coat..

[24] Kattimani V.S., Kondaka S., Lingamaneni K.P. (2016). Hydroxyapatite–Past, Present, and Future in Bone Regeneration. Bone Tissue Regen. Insights.

[25] Wang H. (2004). Hydroxyapatite Degradation and Biocompatibility.

[26] Yang G., Xiao Z., Long H., Ma K., Zhang J., Ren X., Zhang J. (2018). Assessment of the characteristics and biocompatibility of gelatin sponge scaffolds prepared by various crosslinking methods. Sci. Rep..

[27] Panagiotopoulou V.C., Santolini E., Jones E., Jha A., Giannoudis P.V. Adhesives for treatment of bone fractures: A review of the state-of-the art. Injury.

[28] Zysset P.K., Edward Guo X., Edward Hoffler C., Moore K.E., Goldstein S.A. (1999). Elastic modulus and hardness of cortical and trabecular bone lamellae measured by nanoindentation in the human femur. J. Biomech..

[29] Lian H., Zhang L., Meng Z. (2018). Biomimetic hydroxyapatite/gelatin composites for bone tissue regeneration: Fabrication, characterization, and osteogenic differentiation in vitro. Mater. Des..

[30] Alao A.R. (2019). Elasticity, plasticity and analytical machinability prediction of lithium metasilicate/disilicate glass ceramics. J. Mech. Behav. Biomed. Mater..

[31] Alao A.R., Yin L. (2016). Assessment of Elasticity, Plasticity and Resistance to Machining-induced Damage of Porous Pre-sintered Zirconia Using Nanoindentation Techniques. J. Mater. Sci. Technol..

[32] Radtke A., Grodzicka M., Ehlert M., Muzioł T., Szkodo M., Bartmański M., Piszczek P. (2018). Studies on Silver Ions Releasing Processes and Mechanical Properties of Surface-Modified Titanium Alloy Implants. Int. J. Mol. Sci..

[33] Alao A.R., Yin L. (2015). Nanoindentation characterization of the elasticity, plasticity and machinability of zirconia. Mater. Sci. Eng. A.

[34] Menzies K.L., Jones L. (2010). The impact of contact angle on the biocompatibility of biomaterials. Optom. Vis. Sci..

[35] Lin Z., Wang Y., Wang D., Zhao B., Li J. (2013). Porous structure preparation and wettability control on titanium implant. Surf. Coat. Technol..

[36] Tang H., Cao T., Liang X., Wang A., Salley S.O., McAllister J., Ng K.Y.S. (2009). Influence of silicone surface roughness and hydrophobicity on adhesion and colonization of *Staphylococcus epidermidis*. J. Biomed. Mater. Res. -Part A.

[37] Cordero-Arias L., Cabanas-Polo S., Gao H., Gilabert J., Sanchez E., Roether J.A., Schubert D.W., Virtanen S., Boccaccini A.R. (2013). Electrophoretic deposition of nanostructured-TiO_2_/chitosan composite coatings on stainless steel. RSC Adv..

[38] Oliver W.C., Pharr G.M. (1992). An improved technique for determining hardness and elastic modulus using load and displacement sensing indentation experiments. J. Mater. Res..

[39] Mosmann T. (1983). Rapid Colorimetric Assay for Cellular Growth and Survival: Application to Proliferation and Cytotoxicity Assays. J. lmmunol. Methods.

